# Noninvasive assessment of dofetilide plasma concentration using a deep learning (neural network) analysis of the surface electrocardiogram: A proof of concept study

**DOI:** 10.1371/journal.pone.0201059

**Published:** 2018-08-22

**Authors:** Zachi I. Attia, Alan Sugrue, Samuel J. Asirvatham, Michael J. Ackerman, Suraj Kapa, Paul A. Friedman, Peter A. Noseworthy

**Affiliations:** 1 Division of Heart Rhythm Services, Department of Cardiovascular Diseases, Mayo Clinic, Rochester, Minnesota, United States of America; 2 Division of Pediatric Cardiology, Department of Pediatrics and Adolescent Medicine, Mayo Clinic, Rochester, Minnesota, United States of America; 3 Windland Smith Rice Sudden Death Genomics Laboratory, Department of Molecular Pharmacology & Experimental Therapeutics, Mayo Clinic, Rochester, Minnesota, United States of America; Indiana University, UNITED STATES

## Abstract

**Background:**

Dofetilide is an effective antiarrhythmic medication for rhythm control in atrial fibrillation, but carries a significant risk of pro-arrhythmia and requires meticulous dosing and monitoring. The cornerstone of this monitoring, measurement of the QT/QTc interval, is an imperfect surrogate for plasma concentration, efficacy, and risk of pro-arrhythmic potential.

**Objective:**

The aim of our study was to test the application of a deep learning approach (using a convolutional neural network) to assess morphological changes on the surface ECG (beyond the QT interval) in relation to dofetilide plasma concentrations.

**Methods:**

We obtained publically available serial ECGs and plasma drug concentrations from 42 healthy subjects who received dofetilide or placebo in a placebo‐controlled cross‐over randomized controlled clinical trial. Three replicate 10-s ECGs were extracted at predefined time-points with simultaneous measurement of dofetilide plasma concentration We developed a deep learning algorithm to predict dofetilide plasma concentration in 30 subjects and then tested the model in the remaining 12 subjects. We compared the deep leaning approach to a linear model based only on QTc.

**Results:**

Fourty two healthy subjects (21 females, 21 males) were studied with a mean age of 26.9 ± 5.5 years. A linear model of the QTc correlated reasonably well with dofetilide drug levels (r = 0.64). The best correlation to dofetilide level was achieved with the deep learning model (r = 0.85).

**Conclusion:**

This proof of concept study suggests that artificial intelligence (deep learning/neural network) applied to the surface ECG is superior to analysis of the QT interval alone in predicting plasma dofetilide concentration.

## Introduction

Dofetilide, a potent I_Kr_ (hERG; Kv11.1) channel inhibitor, is an effective Class III antiarrhythmic for the maintenance of normal sinus rhythm in patients with atrial fibrillation (AF) [1–3], including those with left ventricular systolic dysfunction[4, 5]. However, its use poses a significant risk of potentially fatal arrhythmia, namely torsades de pointes (TdP), and meticulous monitoring is mandated by the FDA at the time of drug initiation or with changes in dosing. Meticulous monitoring is also critically important for dofetilide reloading after discontinuation of a previously tolerated dose[6].

Dofetilide increases the monophasic action potential duration which is reflected by prolongation of the QT interval on the surface ECG. Therefore, in clinical practice the QT interval is used as a surrogate and essential serves two roles. First, it serves as a surrogate for pharmacologic effect and second, as a marker of potential pro-arrhythmic risk. While QT assessment is the cornerstone of dofetilide dosing adjustments in clinical practice there are a few potential concerns with this approach. The correlation between QTc and plasma concentration is reasonable, but it is not perfectly linear [7, 8], and the relationship is less certain at steady state[9] and at lower heart rates[10] (due to the reverse use dependence properties of the drug). Furthermore, physician measurement of the QT interval is notoriously inconsistent which can lead to inadequate therapy and inappropriate dose titration. Additionally, FDA guidance recommends the QTc must be determined using an average of 5–10 beats, which can be impractical.

Awareness of a patients individual dofetilide level is important for both safety[11] and efficacy[1] particularly as higher doses (and subsequently higher plasma concentration) are associated with greater freedom from AF. The surface ECG contains a wealth of information beyond the QT interval. We and others have demonstrated that computerized analyses can detect subtle T wave changes across a range of cardiovascular diseases and physiologic pertubations[12–16]. While these approaches have been successful, sophisticated and undirected analyses using deep learning (neural networks) have the potential to dramatically extend the power of ECG interpretation [17–19]. The aim of our study was to test the application of deep learning (a convolutional neural network, in this case) to assess morphological changes on the surface ECG (beyond the QT interval) in relation to dofetilide plasma concentrations.

## Methods

### Patient population

**Fig 1:**
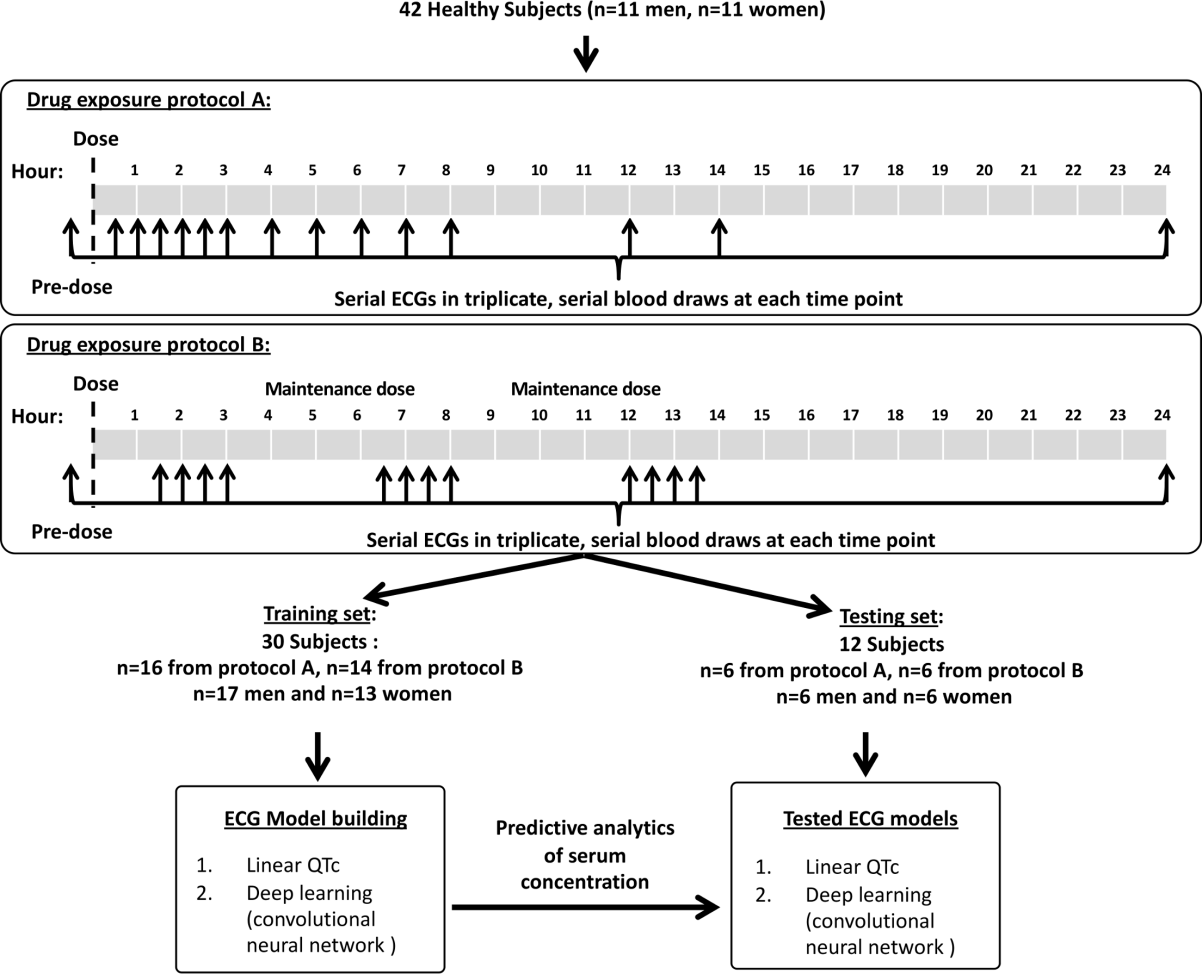
Drug exposure protocol of the randomized controlled trials. For the two models created, 42 patients we used a training cohort of 30 patients with 12 subjects as the testing cohort.

This study population was extracted from a two prospective randomized controlled clinical trials[13, 20]. (**Fig 1**) The data was obtained from PhysioNet[21], a database of recorded biomedical signals and open-source software for analyzing them. The design of the clinical trials has been previously published by the original authors[12, 13, 20]. [Protocol A- https://physionet.org/physiobank/database/ecgrdvq/, Protocol B- https://physionet.org/physiobank/database/ecgdmmld/]

Briefly, the first trial (Protocol A) consisted of 22 healthy subjects, 11 women and 11 men. In the morning of each treatment period, subjects received either dofetilide or placebo. ECGs were recorded at specified time points and a blood sample was drawn for pharmacokinetic analysis after each ECG extraction time point and plasma drug concentration (reported in pg/ml) was measured using a validated liquid chromatography with tandem mass spectroscopy method by Frontage Laboratories (Exton, Philadelphia, PA). The second trial (Protocol B) consisted of 22 subjects (11 women, 11 male) and similar to Protocol A, simultaneous ECGs and blood samples were drawn for pharmacokinetic analysis at specific time points. For this study protocol the subjects were dosed three times during the day. Data was only available on 20/22 patients that received dofetilide.

### ECG recording

Continuous ECGs were recorded at 500 Hz and with an amplitude resolution of 2.5 μV. In Protocol A from the continuous recording, triplicate 10-second ECGs were extracted at predose and 15 predefined time points post-dose (0.5, 1, 1.5, 2, 2.5, 3, 3.5, 4, 5, 6, 7, 8, 12, 14, and 24 h) during which the subjects were resting in a supine position for 10 minutes. In protocol B triplicate 10‐second ECGs were extracted before the draw of each pharmacokinetic sample at 14 predefined time‐points: 1 point pre-dose (-0.5 h) and 13 points post‐dose (1.5, 2, 2.5, 3, 6.5, 7, 7.5, 8, 12, 12.5, 13, 13.5 and 24 h), during which the subjects were resting in a supine position for 10 minutes.

### Model building and predictive analysis

Data from thirty subjects were used for training the model and data from twelve were used for testing. The subjects whose data was used in testing [the last six male and female patients according to study ID numbers] was not used to build any of the algorithms. As highlighted in **Fig 1** we created two models; linear QTc (Bazett’s calculation), and the convolutional neural network, and then used these algorithms to predict the plasma drug concentration on the test samples.

### Deep learning model

**Fig 2:**
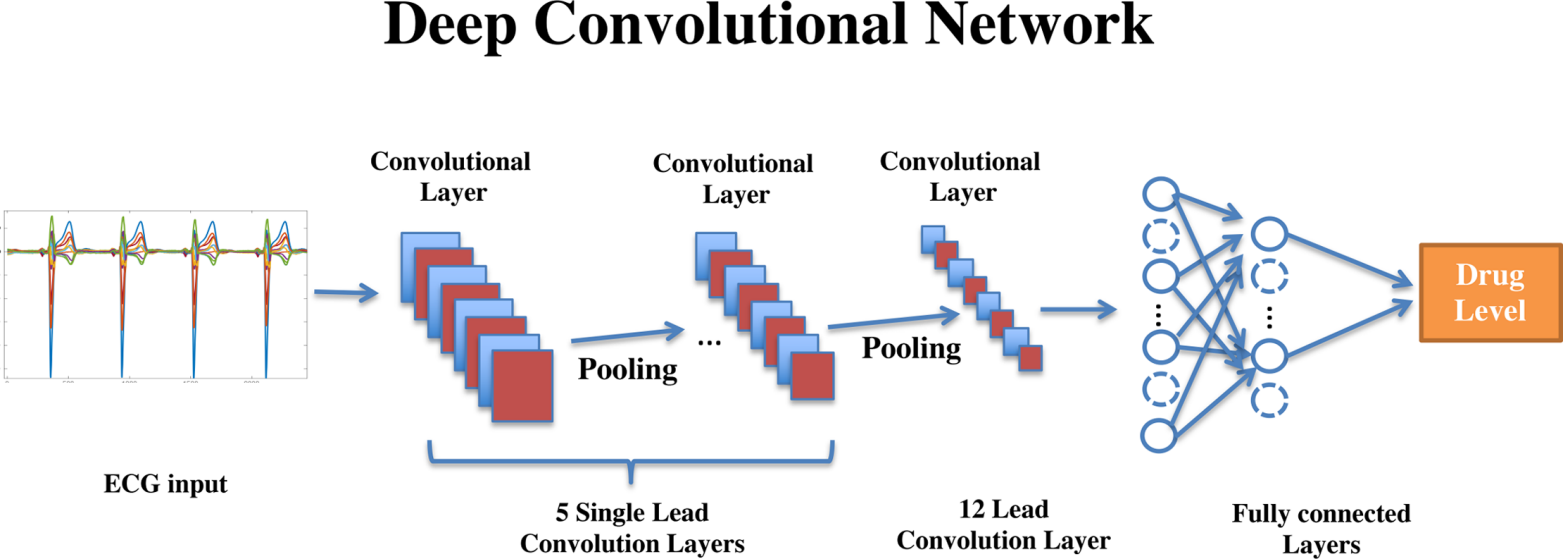
Creation of the Deep Convolutional Network.

We developed and tested a deep learning algorithm that received the raw ECG segment for each sample (*X_i_* = [*x*_*i*1_,*x*_*i*2_…*x_ik_*]) and the matching drug level (*Y_i_*). The goal of the algorithm is to find the optimal nonlinear functions of the signal in an *unsupervised* manner using deep convolutional neural networks and use these as features for a non-linear regression model using a neural network. We used a convolutional neural network with seven convolutional layers followed by two fully connected layers **(Fig 2)**. The first 6 layers are using *n* convolution filters in size of [1xk] with zero padding, where *k* is getting smaller as the layer is deeper and n is getting bigger. A final convolutional layer with 12x1 convolutional filter is combining the features from all of the different ECG leads. Each of the layers is followed by batch normalization[22] and a *ReLu* (Rectified Linear Unit) activation function[23]. The outputs from the last layer in feature extraction network described, is flattened and connected to a fully connected neural network ending in the output neuron, the two hidden layers are with 128 and 64 neurons and are followed with batch normalization and *Relu* activation and dropout with Pr = 0.5. We used the Mean Square Error as the error function with no additional regularization. Model was trained using the Adam optimizer, all weights were initialized using the Xavier method[24]. 10% of training set was used for internal validation and the model with best validation error was saved for testing. (Further information on artificial intelligence models can be read at the following references[25, 26])

### Linear models

As a reference, we trained a linear model based on the QTc calculated from all 12 leads in the training cohort and tested this on the testing cohort. We used the Bazett’s and Hodges formulas for correcting the QT segment for heart rate changes, but the QTc calculated using the Bazett’s was used as it yielded a better model. In this model, the output is a linear function of the QTc. These models were compared to the deep learning model.

### Statistical methods

Categorical variables were expressed as percentages and continuous variables were expressed as mean ± SD. All models were tested on twelve independent patients that were not used for training. From these twelve patients the median value from the triplicate ECG segments matching to one blood draw was used as the final estimated value. This was then tested versus the actual recorded study drug level and mean and standard error was calculated and presented as mean ± SD. An error scatter plot was created for each of the models and a correlation between calculated and actual levels was calculated.

## Results

Forty two healthy subjects (21 females, 21 males) participated in the study with a mean age of 26.6 ± 5.1 years and a mean body mass index of 23.4 ± 2.5 kg/m^2^. The median heart rate across the study was 64 bpm (IQR 57–69). Table 1 shows the QTc at baseline and at peak dofetilide plasma concentration across all testing subjects. S1 Fig highlights the ECGs from the testing cohort at baseline and at peak dosing.

**Table 1:** QTc at baseline and peak dofetilide plasma concentration.

	Patient	Baseline QTc	QTc at Peak Dofetilide Concentration
Training Cohort	1	404.7	449.9
	2	375.4	423.9
	3	447.8	543.8
	4	374.2	441.6
	5	430.9	490.7
	6	424.3	525.3
	7	394.9	514.1
	8	379.1	445.3
	9	368.2	427.6
	10	381.1	464.2
	11	373.9	511.4
	12	380.1	413.8
	13	392.4	458.3
	14	352.9	434.2
	15	358.6	440.3
	16	406.7	463.8
	17	396.2	442.5
	18	421.4	453.2
	19	407.7	402.8
	20	403.3	422.1
	21	397.1	426.8
	22	382.5	420.4
	23	389.4	399.8
	24	387.4	439.7
	25	421.5	469.0
	26	408.8	518.8
	27	391.4	412.5
	28	404.5	431.8
	29	371.4	408.7
	30	387.1	438.7
Testing Cohort	1	389.8	465.9
	2	392.4	425.9
	3	410.1	461.3
	4	372.6	474.6
	5	410.2	481.9
	6	394.5	433.5
	7	412.1	452.0
	8	437.6	498.0
	9	368.2	426.5
	10	426.5	457.7
	11	373.0	401.2
	12	401.8	418.7

### Correlation with drug level

**Fig 3:**
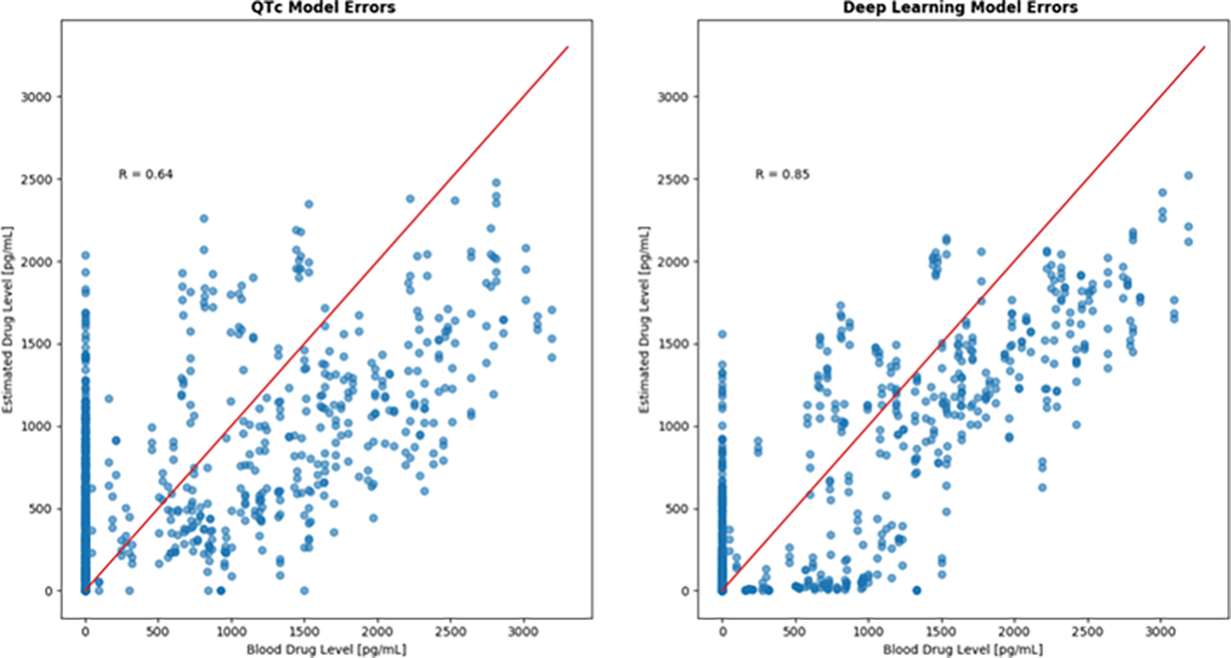
Correlation of the QTc model and the Deep Neural Network (Deep Convolutional Network).

**Fig 3** shows the correlation between the models and the dofetilide drug concentration. The linear QTc model, showed a reasonable correlation (r = 0.64). The best correlation was found with the deep learning model which strongly and accurately correlated with the plasma dofetilide drug concentration (r = 0.85).

**Fig 4:**
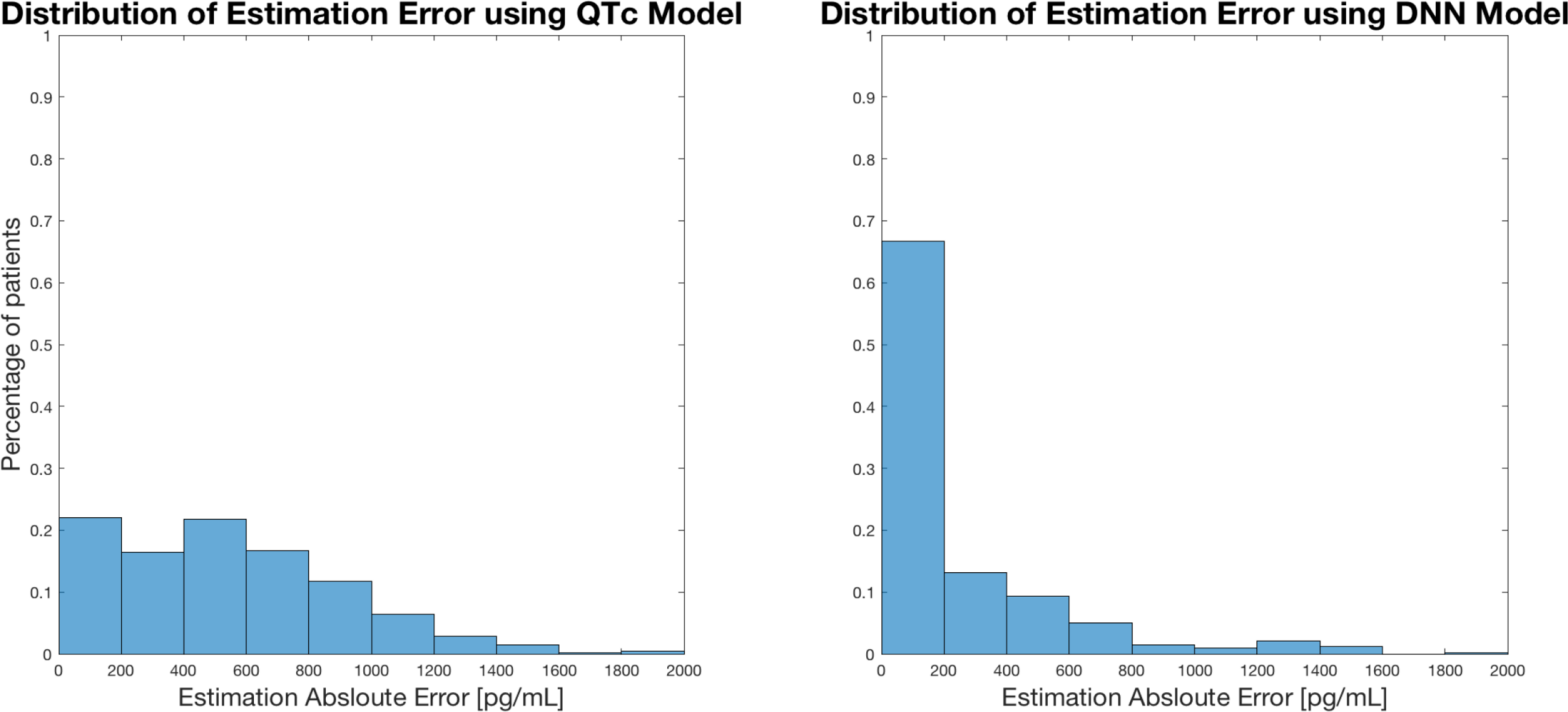
Histograms showing the distribution of drug error with the models. DNN—Deep Neural Network (Deep Convolutional Network).

**Fig 4** shows the error in drug level calculated from the ECG using the blood test as the gold standard from each of the ECG models. For the QTc model the error was 487 ± 432 pg/ml. The smallest error was observed using the deep learning model, 296 ± 347 pg/ml.

**Fig 5:**
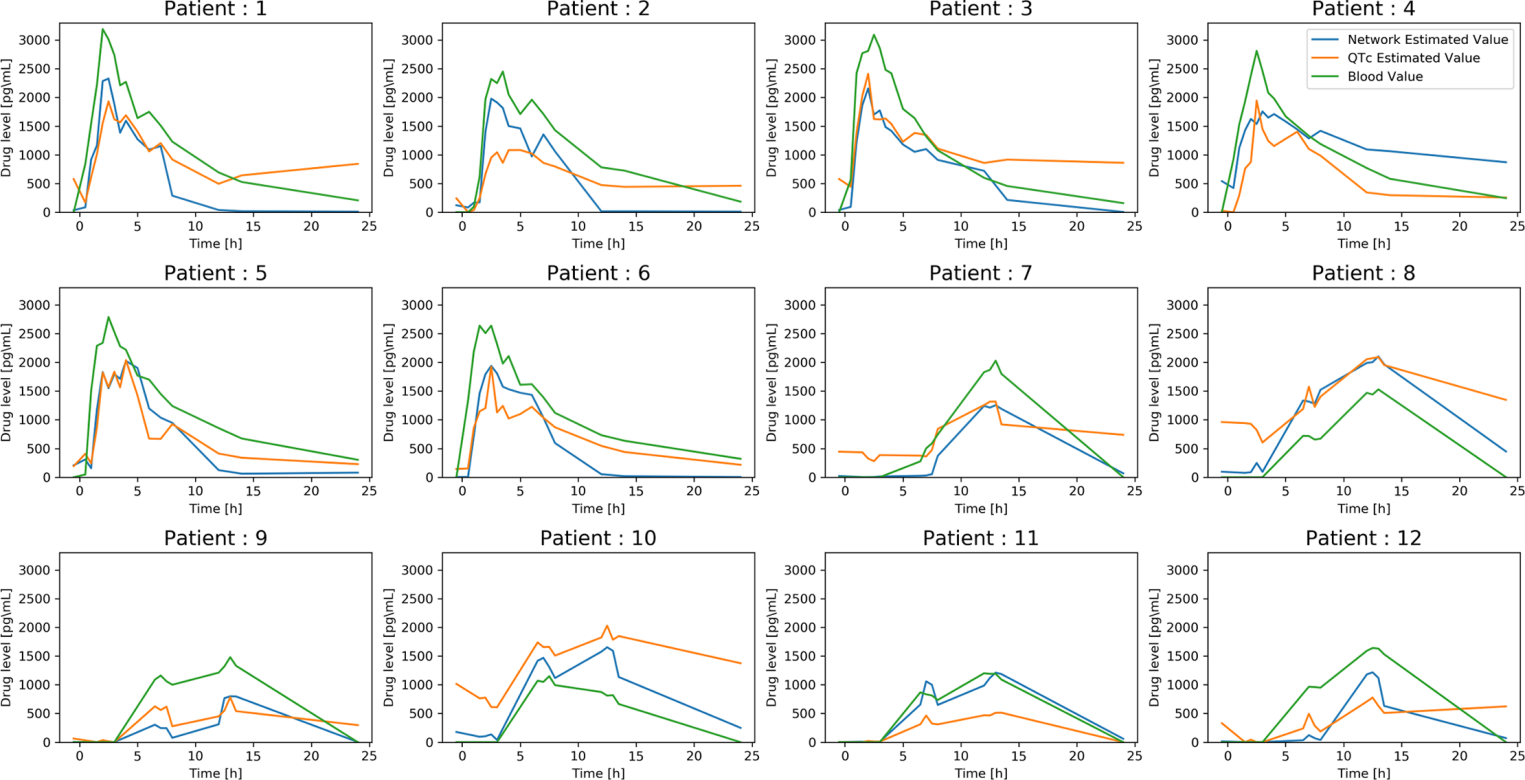
The drug level is shown on the ordinate, with time depicted on the abscissa. The red line indicates the recorded drug levels, whereas the blue line (DNN) and orange line (QTc) indicate the ECG-predicted drug level for each of the models. DNN—Deep Neural Network (Deep Convolutional Network).

For **Fig 5** the drug level, at each time point averaged across the triplicate ECGs, is shown on the ordinate, with time depicted on the abscissa. The red line indicates the drug level, whereas the blue line (deep learning network) and orange line (QTc) indicate the ECG-predicted drug level for each of the models.

## Discussion

This study, to the best of our knowledge, is the first application of artificial intelligence (deep learning with a convolutional neural network) to the surface ECG to determine plasma drug concentrations. As a proof of concept study, we demonstrate the potential power of this technology when applied to the surface ECG. We found that deep learning resulted in better correlation, smaller error, and more accurate prediction of plasma dofetilide concentration than the QTc. Knowledge of a patients drug concentration is important for both safety and efficacy of dofetilide dosing,[1, 11] yet in current clinical practice levels are rarely measured due to their cost and delay in results. Instead, we rely on the QTc interval which is an imperfect surrogate both in terms of its relationship to plasma concentration and issues with calculation/measurement.

### QTc and dofetilide concentration

The relationship between QTc and dofetilide concentration is reasonable however there have been some concerns raised. Early work by Sedgwick[8] demonstrated reasonable correlation (r = 0.81) but these studies did not evaluate the full range of concentrations and may have falsely predicted a linear relationship. Indeed, other studies have suggested a sigmoidal drug level- QTc relationship[27] with some studies demonstrating a significant impact of heart rate on this relationship, suggesting a weak correlation at low doses or at higher heart rates[10]. Other investigators have also demonstrated remarkable inter-individual variation in the QT response to dofetilide [28]. Additionally, the QTc response to plasma dofetilide concentration is reported to be greater after the first dose than at steady state.

Another major concern is the measurement of the QT interval itself is notoriously heterogenous[29–31]. Errors in QT interval measurement can have significant clinical consequences and result in under- or over-dosing, or even stopping of the drug[32]. The choice of ECG lead,[33] method of heart rate correction,[34] and determination of the end of the T wave (particularly when U waves are preset) are common sources of variability[31]. The FDA has recommended assessing the QTc as an average over 5–10 beats which can be impractical, and clinicians often rely on the automated QT interval calculations[35, 36].

While it is clear the QT measurement is imperfect and fraught with potential concerns, it remains very much entrenched in clinical practice. We believe systematic evaluation of the ECG, perhaps with a neural network approach as demonstrated here, may refine the current approach to repolarization assessment and holds great promise in refining our ability to noninvasively monitor drug levels and potentially efficacy and pro-arrhythmic risk[37].

### Deep learning with convolutional neural networks

Machine learning is essentially a method of artificial intelligence that can identify relationships within data without prior definition[38]. Deep learning takes this one step further, in that it allows computational models that are composed of multiple processing layers based on neural networks to learn from representations of data with multiple levels of abstraction[39]. Deep learning is a relatively new approach, but it has shown promise in data synthesis in many health care applications. Neural networks have been employed for complex tasks in cardiology[40], particularly the classification of constrictive pericarditis and restrictive pericarditis,[41] the discrimination of physiological versus pathological patterns of hypertrophic remodeling,[17] risk stratification and prognosis in heart failure,[18] and a variety of ECG interpretation applications[19, 42, 43]. We have extended the current applications of deep learning, with a proof of concept using the surface ECG to identify drug levels. Importantly, inherent to deep learning, the segments of the ECG (QRS, T wave, etc) actually used by the computer are not known; the entire signal was fed to the network and it learned the predictive model via iterative observations.

### Future directions

The ability to determine plasma drug concentration noninvasively, reproducibly, remotely, and without complex and expensive equipment hold great promise for significant safety and efficacy benefits. In our case, since the drug level is ECG derived, it is not dependent on repeated phlebotomy, permitting continuous drug level assessment as opposed to intermittent and time-delayed results. Moreover, expensive equipment and reagents required for blood analysis are avoided. Furthermore, this approach may augment drug safety analysis as part of the ‘thorough QT study’ required by the FDA. Beyond the realm of drug dosing and safety, deep learning applied to the surface ECG may be useful in other clinical situations that aim to assess repolarization or arrhythmic risk including long QT syndrome (particular with concealed phenotypes), sudden cardiac death risk prediction, and cardiac screening prior to athletic participation, to name a few.

### Limitations

Although the data for this study are of high quality and were meticulously collected, the population includes only a small sample of healthy volunteers. In order to enable broad clinical application of this approach, further study in patients with a range of physiologic states (e.g. variations in dosing, heart rates) and underlying cardiovascular substrates is needed. We did not have data to permit assessment of arrhythmic risk, so we cannot comment on the use of our neural network for that. This would be an important area of future research. Additionally, we used Bazett’s formula to calculate the QTc, which is the most commonly used in clinical practice. However, there is the possibility of overestimation of repolarization at higher heart rates and underestimation at lower heart rates. It has been described that single nucleotide polymorphisms (SNPs) can affect the QT interval in the general population and in those receiving dofetilide, unfortunately since we do not have access to stored blood samples we were unable to assess the relationship between these variants and QT drug-response. Last, the application of this data to those on long-term therapy with dofetilide is unknown as we only had data from volunteers receiving a single oral dose.

## Conclusion

This proof of concept study demonstrates that artificial intelligence applied to the surface ECG provides an innovative method to assess plasma drug concentrations. With further study, this novel approach in the future may have the potential to significantly impact clinical practice by providing inexpensive, point of care real-time drug levels.

## Supporting information

S1 Fig ECG tracings from testing cohorts’ baseline and at peak dofetilide level. (PDF)

## Abbreviations

ECG: electrocardiogram
TdP: torsades de pointes
