# Supplementary figures and images for: Noninvasive assessment of dofetilide plasma concentration using a deep learning (neural network) analysis of the surface electrocardiogram: A proof of concept study
Source: PLoS One. 2018 Aug 22;13(8):e0201059. doi: 10.1371/journal.pone.0201059 (PMC6104915; doi:10.1371/journal.pone.0201059)

S1 Fig

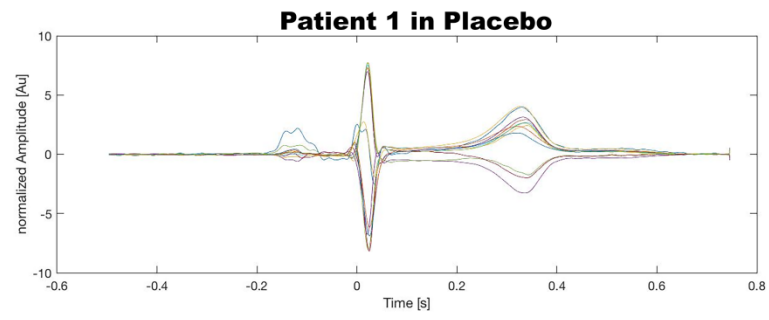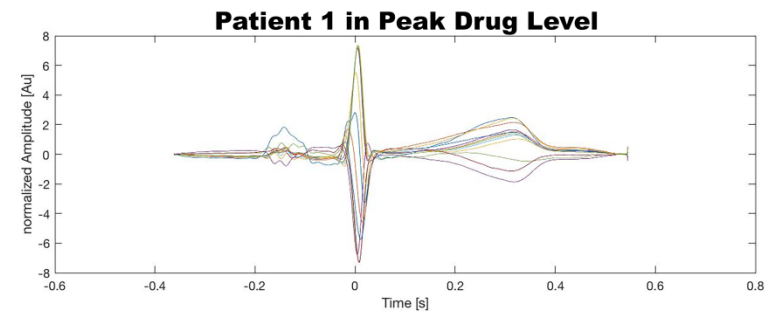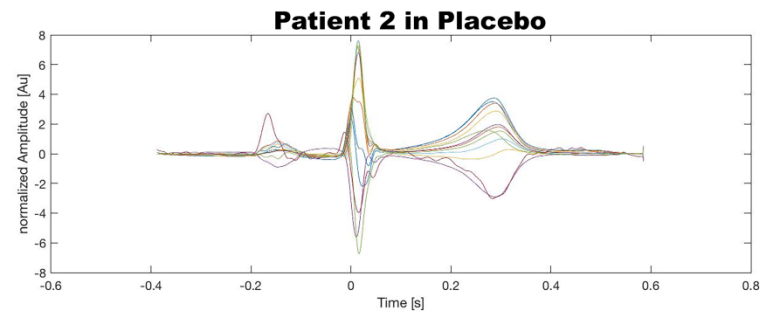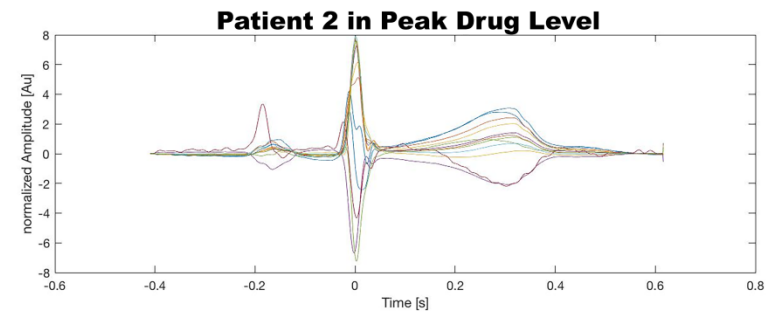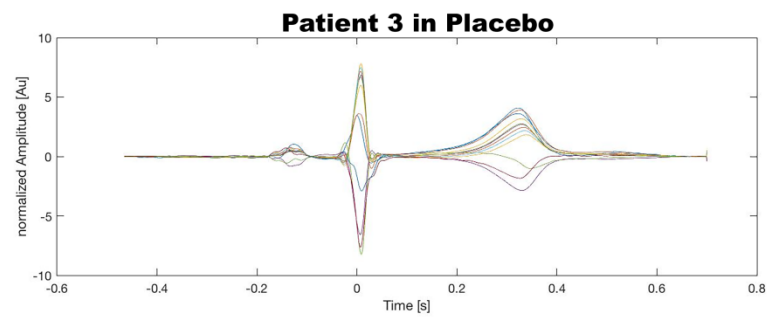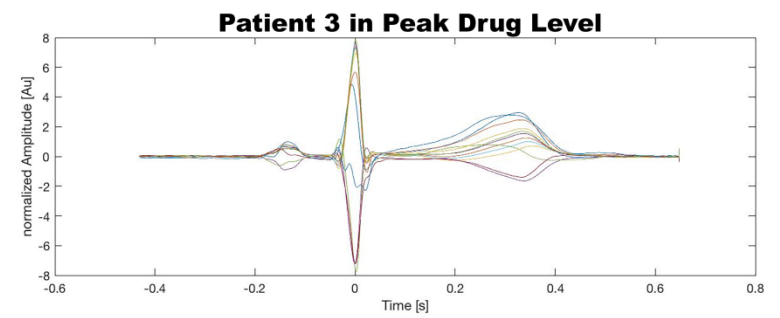

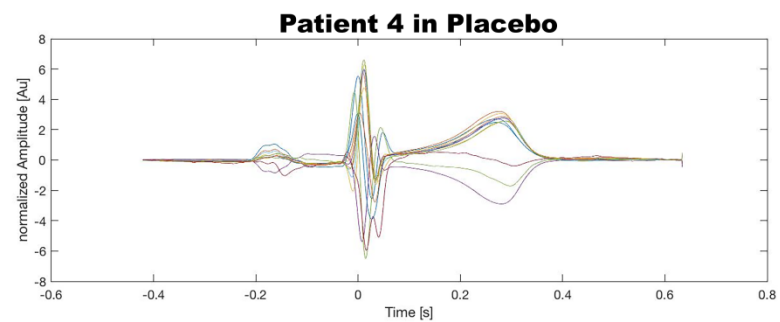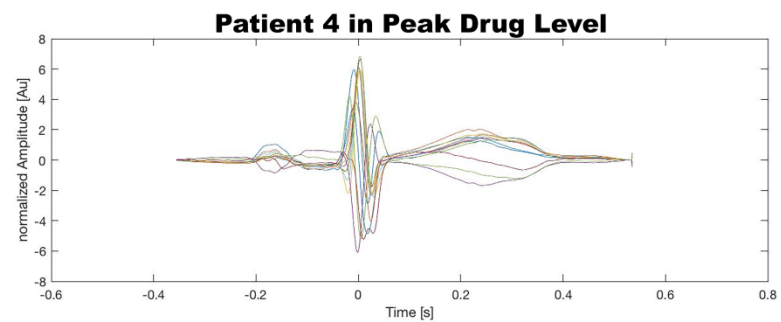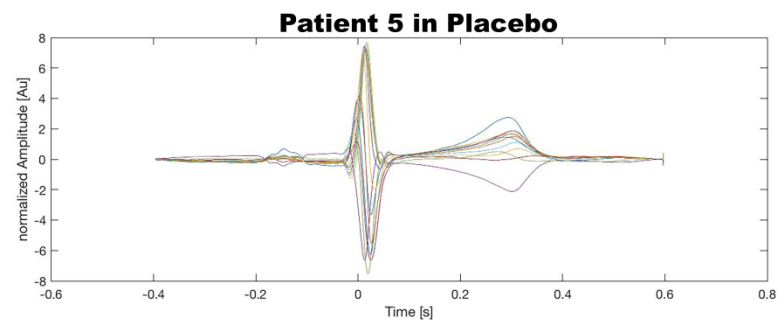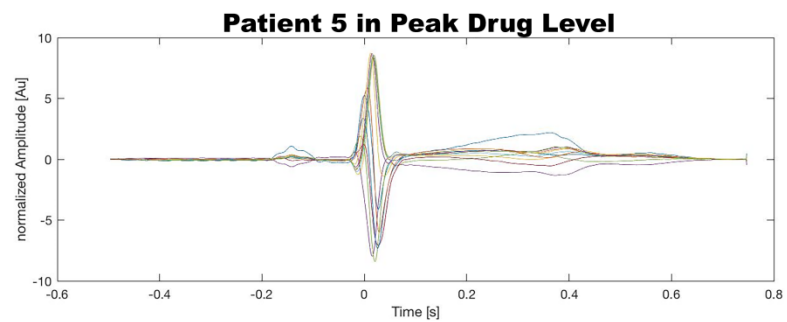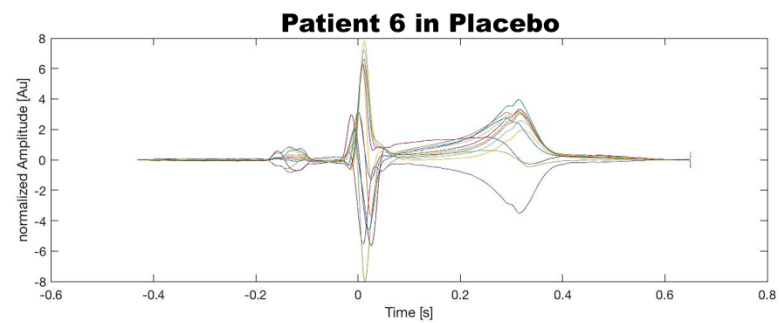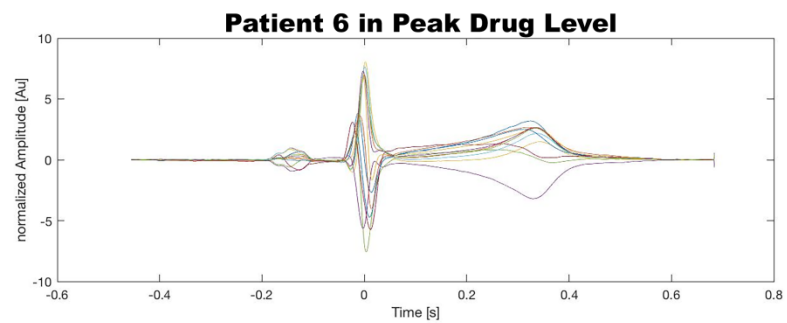

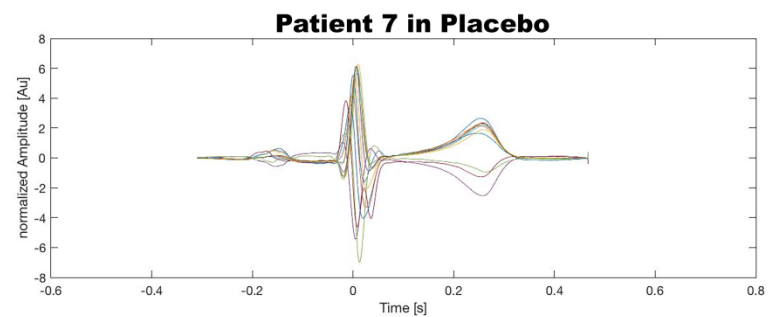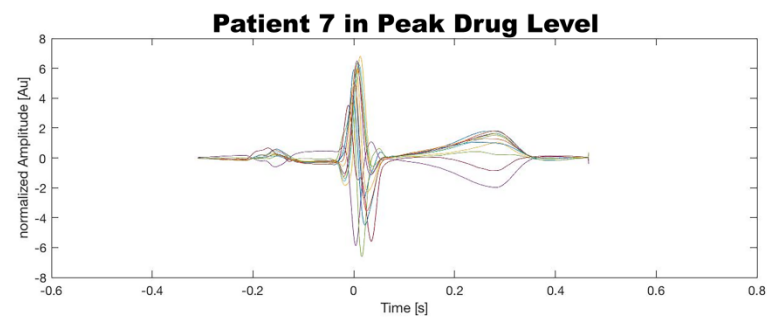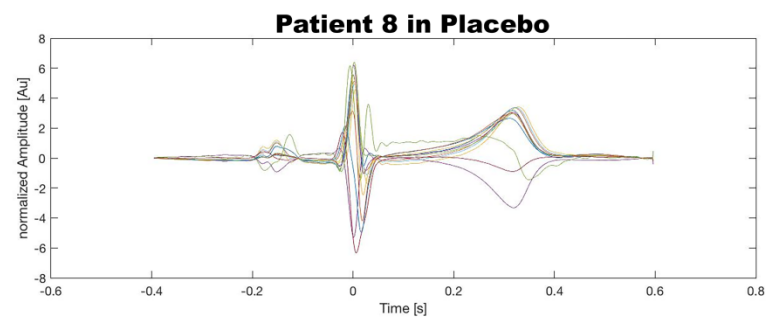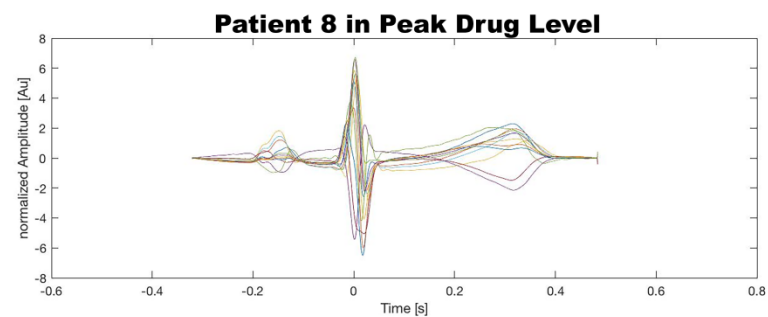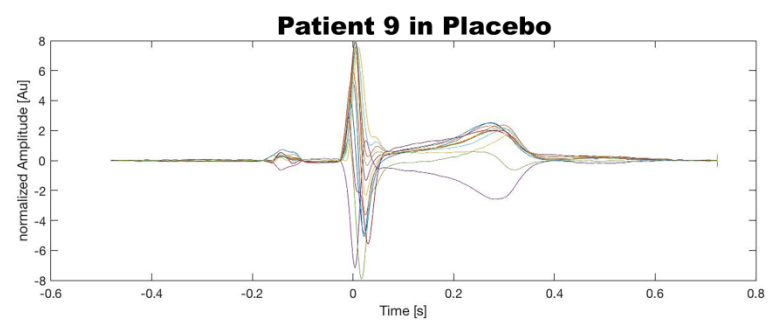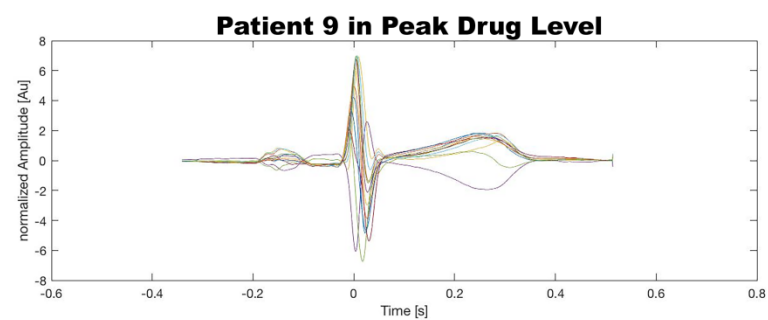

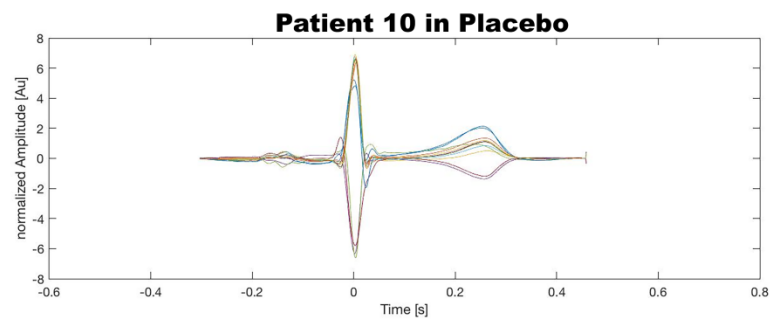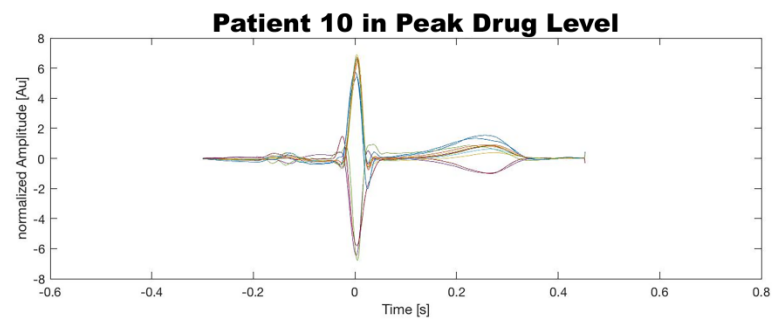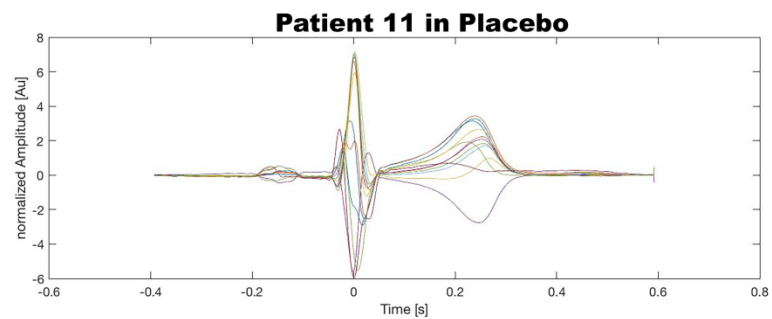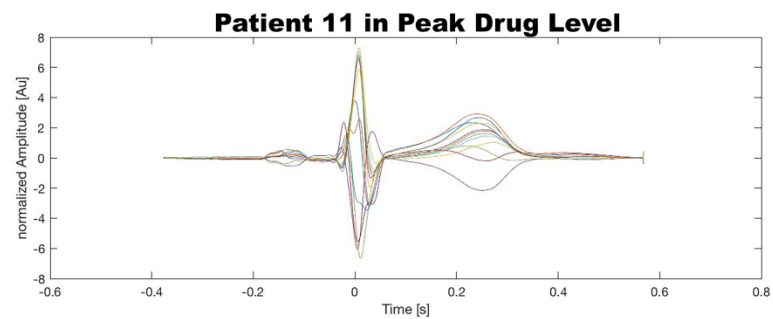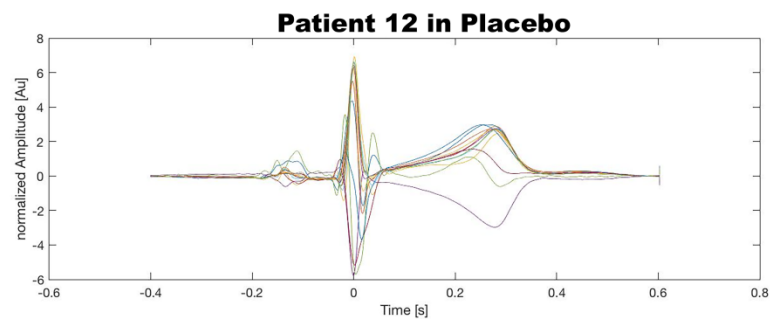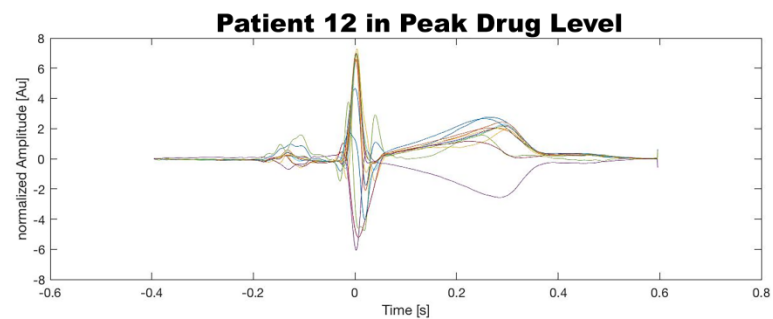

Supplement: S1 Fig — (PDF) [file pone.0201059.s001.pdf]
